# The Genetics of Parkinson’s Disease and Implications for Clinical Practice

**DOI:** 10.3390/genes12071006

**Published:** 2021-06-30

**Authors:** Jacob Oliver Day, Stephen Mullin

**Affiliations:** 1 Faculty of Health, University of Plymouth, Plymouth PL4 8AA, UK; jacob.day@plymouth.ac.uk; 2 Department of Clinical and Movement Neurosciences, University College London Institute of Neurology, London WC1N 3BG, UK

**Keywords:** Parkinson’s disease, genetics, precision medicine, clinical trials, monogenic, polygenic

## Abstract

The genetic landscape of Parkinson’s disease (PD) is characterised by rare high penetrance pathogenic variants causing familial disease, genetic risk factor variants driving PD risk in a significant minority in PD cases and high frequency, low penetrance variants, which contribute a small increase of the risk of developing sporadic PD. This knowledge has the potential to have a major impact in the clinical care of people with PD. We summarise these genetic influences and discuss the implications for therapeutics and clinical trial design.

## 1. Introduction

Parkinson’s disease (PD) is a neurodegenerative condition affecting over 6 million people worldwide that is expected to double in prevalence by 2040 [1]. It is characterised by a core set of movement (motor) abnormalities - slowness of movement, muscle rigidity and tremor – as well as a number of non-motor features such as constipation, anxiety and dementia [2]. There is often a prodromal phase of non-motor symptoms which precede motor symptoms by many years [3]. The pathological hallmark of PD is Lewy bodies, which are aggregates of misfolded α-synuclein protein (encoded by the *SNCA* gene) which lead to the loss of midbrain dopamine-producing neurons. However, diagnostic criteria for PD are based on clinical features alone [4,5]. PD can therefore be considered a clinical syndrome with aetiological pathways that converge onto a common final presentation of dopamine loss and clinical Parkinsonism. There are currently no disease-modifying therapies for PD and treatment focusses on dopamine replacement to alleviate symptoms. 

The exact cause of PD is unknown. Early twin studies showed only a slight excess of PD in monozygotic compared to dizygotic twins and led to the conclusion that PD was an exclusively acquired condition [6]. However, the discovery of a familial form of PD due to a single gene mutation [7] and later twin studies incorporating a longitudinal design [8,9] have led to the current paradigm of PD as a complex disease with both genetic and environmental contributions [10]. Meta-analysis suggests that the presence of a family history of Parkinson’s confers a 3–4× increase in PD risk, implying a significant effect of shared genetic and environmental factors to sporadic PD risk [11].

Age is known to be the greatest risk factor for developing PD [12]. The age-related decline in midbrain dopamine-producing neurons may reach a threshold below which clinical symptoms emerge and an individual’s total number of dopaminergic neurons may therefore contribute to the risk and age of onset of PD [13]. A number of environmental exposures have also been associated with PD in observational studies including head trauma, dairy consumption and pesticide exposure [14]. Cigarette smoking and caffeine consumption have been associated with reduced risk of PD, although the biological mechanisms for this remain unclear [15,16].

**Figure 1:**
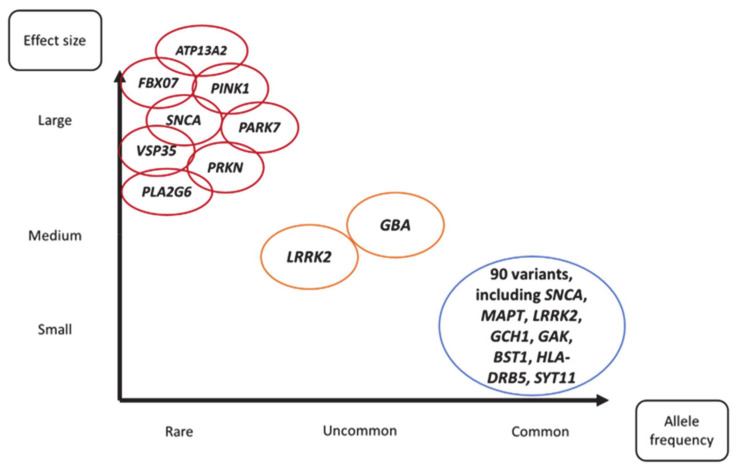
Summary of genetic variants in Parkinson’s disease grouped according to allele frequency and associated risk of Parkinson’s disease.

Genetic variation is estimated to contribute approximately 25% to the overall risk of developing PD [9,17,18]. The genetic variants related to PD vary in terms of frequency and risk of PD (Figure 1). On the one hand, there are a number of rare variants in single genes that are sufficient to cause disease (i.e., are pathogenic). These monogenic causes of PD were predominantly identified through linkage analysis of affected families. Examples of such genes include *SNCA*, *PARK7* and *PRKN.* On the other hand, genome wide association studies (GWAS) have identified large numbers of common genetic variants that individually contribute a small amount to the risk of developing PD. In the middle of this spectrum lie variants that are uncommon (but not rare) and exert an intermediate risk, such as *GBA* and *LRRK2* variants. 

Genetics provides one way in which PD can be subdivided. Different subgroups may have separate aetiologies, treatments and prognosis [19]. Common stratification factors for PD include age of onset (early- vs late-onset PD, typically with a cut-off of 50 years of age), the presence or absence of family history (familial vs sporadic PD) and the presence of pathogenic variants (monogenic vs idiopathic PD). Truly monogenic forms of PD are very rare and may not represent the same disease processes that occur in idiopathic PD. For instance, *PRKN*, *PINK1* and *PARK7* are all genomic regulators of mitochondrial function [20]. This is important to consider as the majority of laboratory models have focussed on pathways that were identified by monogenic causes of PD [21]. Encouragingly, some of the common variants identified in GWAS also implicate genes that cause monogenic PD [21]. For instance, common variants at the *SNCA* loci exert the largest contribution to PD risk in GWAS and rare *SNCA* variants are a cause of monogenic PD, supporting the existing α-synuclein centric model of PD [18]. Conversely the *MAPT* loci encodes tau, aggregates of which are found in a range of neurodegenerative diseases such as Alzheimer’s disease, frontotemporal dementia and progressive supranuclear palsy, suggesting the involvement of other pathways in PD pathogenesis [18,22].

An understanding of the genetic factors influencing a disease process can have major impacts on clinical care. Firstly, it can identify causative genes and highlight biological pathways important to pathogenesis. This in turn can allow the development of therapeutics which target those biological pathways. Secondly, it can allow diverse presentations of disease to be accurately subdivided into groups of shared genetic causes. This is essential for ‘precision medicine’, where management strategies are focussed on an individual’s specific disease subtype. Finally, greater knowledge of the effect of genetic variants on the risk, onset and progression of disease means the implications and prognosis can be discussed frankly with individuals, who are then empowered to make educated decisions. 

This review will first summarise the monogenic and polygenic variants associated with PD and then discuss the clinical implications of this knowledge. We shall also draw parallels with diabetes where appropriate to highlight how this knowledge may shape and influence disease management.

## 2. Monogenic Parkinson’s Disease

A range of loci and genes have been associated with PD phenotypes in a Mendelian fashion and were originally designated a ‘*PARK*’ locus with the number representing the chronological order of discovery. However, multiple *PARK* loci can refer to the same gene (e.g., *PARK1* and *PARK4* both refer to *SNCA*) and some early *PARK* loci are no longer thought to be disease-causing (e.g., *PARK5*). Current recommendations are to use gene names in preference to numbered loci [23], although it is important to be aware of this previous nomenclature and the alternative gene names are listed in Table 1 for clarity.

Variants with high penetrance are very rare whereas those with variable penetrance are more common worldwide, particularly in specific populations. Table 1 summarises these genes, including those historically associated with PD pedigrees in which subsequent conflicting reports and lack of segregation mean the evidence for pathogenicity is low.

### 2.1. Single Genes with High Penetrance

#### 2.1.1. SNCA

The first monogenic cause of PD was discovered in a large Italian family with an autosomal dominant pattern of PD presenting with typical clinical features but early onset (median age 46 years). Linkage studies identified an area of chromosome 4q that segregated with the disease [24]. Candidate gene sequencing of this region subsequently demonstrated a missense variant (c.209G>A) in the α-synuclein gene (*SNCA*) which causes an amino acid substitution (p.A53T) [7]. The same missense variant was found in unrelated Greek pedigrees. Subsequent studies of familial and sporadic PD cohorts suggest it is an extremely a rare cause of PD overall [25,26]. Other missense *SNCA* variants have since been discovered including p.A30P [27], p.E46K [28] and p.G51D [29]. Duplications and triplications of *SNCA* can also cause PD with evidence for a ‘dosage effect’, where greater expression of α-synuclein leads to more severe clinical features [30,31]. However, pathogenic *SNCA* variants only account for a tiny proportion of PD cases [32,33,34].

α-synuclein is highly expressed in the brain and localises predominantly to pre-synaptic terminals of neurons. The discovery that α-synuclein is the major component of Lewy bodies identified a link between the genetic and pathological features of PD [35]. A range of laboratory and animal studies have established that α-synuclein can form abnormal aggregates which are neurotoxic [36,37,38]. α-synuclein aggregates are able to spread within the central nervous system with the fibril form being particularly toxic. Different clinical features are observed depending on the form and location of abnormal α-synuclein aggregates [39]. This aggregation process is accelerated by *SNCA* pathogenic variants or by an increase in intracellular alpha synuclein concentration disrupting proteasome function and exacerbating oxidative stress [40,41]. 

Despite the rarity of pathogenic *SNCA* variants, the relevance of α-synuclein to both monogenic and idiopathic forms of PD has been established by the presence of Lewy bodies, the altered expression of *SNCA* in neurons of people with idiopathic PD [42] and the *SNCA* loci identified in GWAS studies of idiopathic PD (see Section 3.). A number of overlapping functions for α-synuclein have been proposed with interactions demonstrated with other monogenic PD gene products and involvement in multiple pathways including synaptic vesicle function, lysosomal function, mitochondrial function and inflammation [43]. Therefore the biological pathways of α-synuclein aggregation have become the central hypothesis for the molecular processes causing PD.

#### 2.1.2. VPS35

Independent exome sequencing studies in 2011 identified a missense variant in the *VPS35* gene (p.D620N) of Swiss [44] and Austrian [45] families with an autosomal dominant pattern of PD that cosegregrated within the family and was not present in healthy controls. *VPS35* encodes a component of the multimeric retromer complex which mediates trafficking of endosomes. It appears to be important in neuronal transport to dendrites [46] and neuronal cells from humans with the p.D620N variant show disrupted endosomal transport and abnormal accumulation of α-synuclein and reactive oxygen species [47,48]. Although pathogenic variants in *VPS35* are particularly rare, comprising only 0.2% of European patients with suspected autosomal dominant PD, patients manifest typical PD symptoms with a good response to L-DOPA [49,50]. It is reasonable to consider that the gene function, particularly with its association with α-synuclein and mitochondrial function, is also implicated in sporadic PD.

#### 2.1.3. PRKN/ PARK7/ PINK1

These genes will be considered together as their protein products are linked to mitochondrial function and biallelic variants all cause autosomal recessive forms of PD. Between 1998 and 2004, studies of families, often consanguineous, affected by early-onset forms of PD with a suspected autosomal recessive inheritance pattern identified biallelic loss of function variants in *PRKN* (previously called *Parkin*) [51], *PARK7* (previously called *DJ-1*) [52] and *PINK-1* [53]. *PRKN* encodes Parkin which is a ubiquitin ligase involved in the proteasomal degradation system [54] and also has a role in the maintenance of mitochondrial structure and DNA integrity [55]. *PARK7* encodes the DJ-1 protein (or Parkinson disease protein 7) whose specific function is unclear but has been shown to interact with a number of proteins including tau [56], Parkin and PINK1. The product of *PINK1*, PTEN-induced kinase 1, is a mitochondrially associated kinase that may have an anti-inflammatory role in animal models [57]. Parkin, DJ-1 and PINK1 form a ubiquitin ligase complex whose function is impaired by pathogenic variants [58]. Taken together, the evidence suggests that Parkin, DJ-1 and PINK1 interact within the ubiquitin-proteasome system and have a role in the maintenance of mitochondrial structure. Autopsy studies have shown variable results with respect to Lewy body pathology in these genetic forms of PD. In particular, initial studies demonstrated a lack of Lewy bodies in patients with biallelic *PRKN* mutations, questioning its relevance to sporadic PD [59,60,61]. However, subsequent case reports have identified Lewy bodies in homozygous and heterozygous cases of *PRKN*-PD [62,63].

#### 2.1.4. Others

A number of other genes have been implicated in monogenic PD (see Table 1) but either result in a complex phenotype with significant features in addition to Parkinsonism or have less robust evidence for pathogenicity. 

*ATP13A2* biallelic variants result in adolescent-onset PD with dementia and hallucinations [64]. The protein is involved in lysosomal function [65]. Biallelic variants in the *FBXO7* gene have been associated with an early-onset form of PD with pyramidal signs [66,67,68] and FBXO7 interacts with PINK1 to enable its degradation [69]. Biallelic variants in the phospholipase-encoding *PLA2G6* gene were originally described in neurodegeneration with brain iron accumulation but can also cause autosomal recessive PD with or without cognitive, pyramidal and dystonic features [70,71,72]. *DNAJC6* and *SYNJ1* encode interacting proteins involved in synaptic vesicle recycling and result in early-onset autosomal recessive forms of PD [73,74]. *DNAJC13* and *TMEM230* also have a role in synaptic vesicle processing, but variants were discovered in the same affected PD pedigree and their pathogenicity remains uncertain [75]. 

Some genes have only been reported in single pedigrees plus isolated cases (e.g., *TMEM230* variants [75,76]) or have conflicting presence in healthy controls (e.g., *UHCL1*, *HTRA2* [77]). These could represent variants with reduced penetrance or simply erroneous associations with disease.

### 2.2. Single Genes with Variable Penetrance

*LRRK2* and *GBA* have been associated with familial PD. *GBA* in particular does not tend to demonstrate a Mendelian (i.e., autosomal recessive or dominant pattern) of inheritance. They can either be considered as genetic ‘risk factors’, where the presence of certain variants confers an increased risk developing PD, or as autosomal dominant pathogenic variants with incomplete penetrance. The distinction is somewhat arbitrary, particularly when different variants are considered, as some display high penetrance and could be considered monogenic causes of PD.

#### 2.2.1. LRRK2

Heterozygous missense *LRRK2* variants were originally identified in families with autosomal dominant patterns of PD inheritance [78,79]. Subsequent work has identified the c.6055G>A variant (p.G2019S) as the most common pathogenic variant worldwide. It is particularly common in Ashkenazi Jewish and North African Berber populations with prevalence rates, respectively, as high as 26% and 41% in some cohorts. The risk of PD by aged 80 years in carriers of the p.G2019S variant is estimated to be 25–74% [80,81,82]. A number of other pathogenic *LRRK2* variants have been described. Some have been described through linkage studies in kindreds with Parkinson disease with an autosomal dominant pattern of inheritance, whilst other appear to exert marginal increases in PD, characterised by genetic case control data [83,84]. This hints at significant heterogeneity of PD risk associated with individual *LRRK2* variants, although our understanding of this is certainly not as developed as in the case of *GBA*. 

Both environmental (e.g., cigarette smoking, non-steroidal anti-inflammatories) and genetic modifiers have been proposed as factors to explain this incomplete penetrance [85,86,87]. Penetrance of *LRRK2*-PD varies between Arab Berbers and Norwegians, whereas age of onset of idiopathic PD does not, suggesting disparate genetic and environmental modifiers in *LRRK2*-PD penetrance [88]. Conversely, some studies have suggested a lower penetrance amongst Ashkenazi Jewish p.G2019S subjects, although this difference in penetrance may reflect varying degrees of ascertainment bias (i.e., where carriers were identified on this basis of being blood relatives of an index case with PD and may carry genetic cofactors that increase PD risk) [81]. The PD polygenic risk score modifies *LRRK2*-PD penetrance and 1 significant individual locus has been identified that modifies penetrance (within the *CORO1C* gene) [89]. The risk of PD varies according to the specific *LRRK2* variants and certain variants, such as p.R1398H, appear to exert a protective effect [90].

The clinical phenotype of *LRRK2*-PD is broadly similar to that of idiopathic PD with a good response to L-DOPA and a median age of onset of 57 years. However, there is contradictory evidence as to the specific phenotype of *LRRK2*-PD. Some studies suggest that p.G2019S *LRRK2* variants may have higher rates of motor complications [83,91]. One large study suggests a more benign clinical course with a tremor predominant phenotype [82], although other studies do not replicate this finding [81]. The identification of *LRRK2* missense variants in ~1% of sporadic PD and *LRRK2* loci in GWAS, suggests that *LRRK2* has an important role in idiopathic as well as *LRRK2*-PD.

*LRRK2* encodes the leucine-rich repeat kinase 2 protein which has both kinase and GTPase domains. Its physiological role is thought to include autophagy, mitochondrial function and microtubule stability [92]. Pathogenic variants lead to increased kinase domain activity, raising the tantalising possibility of pharmacological inhibition as a possible therapeutic strategy [93,94]. Moreover, LRRK2 kinase activity is enhanced in post-mortem brain tissue of people with idiopathic PD who lack *LRRK2* variants [95]. Despite animal models suggesting off target effects of LRRK2 inhibition [96,97], the apparent benign presence of heterozygous loss of function *LRRK2* variants in humans is reassuring that partial reduction in LRRK2 function is safe [98,99].

#### 2.2.2. GBA

Biallelic (i.e., homozygous or compound heterozygous] variants in the *GBA* gene can lead to Gaucher disease, a lysosomal storage disorder caused by reduced activity of the *GBA*-encoded enzyme glucocerebrosidase (GCase). Its relevance to PD was first considered when patients with Gaucher disease were noted to develop disproportionate levels of Parkinsonian features [100]. The observation of high rates of PD in the families of those with Gaucher disease suggested that heterozygous *GBA* variants cause PD independently [101]. Case-control studies across various populations confirmed an increased risk of PD in those with *GBA* variants [102]. 

Unhelpfully, two nomenclatures which include or omit a 39 amino acid leader of the *GBA* gene exist. Here we refer to the more commonly used historical classification, with the newer classification (which complies with HGNC guidelines) in brackets. 

The range of *GBA* variants identified (to date over 200 Gaucher causing *GBA* variants have been documented [103,104])complicates estimation of PD risk. High quality prospective data amongst those with a diagnosis of Gaucher disease suggest that by 80 years the penetrance of PD is approximately 10% [105]. As with LRRK2, it is likely that ascertainment bias has inflated risk estimates of PD penetrance in at least some cohorts [106,107]. Overall genetic case control data suggests that eleven [predominantly missense] individual *GBA* variants increase PD risk [108]. p.N370S (p.N409S) and p.L444P (p.L483P) are the most common worldwide, with the p.N370S (p.N409S) variant particularly seen in Ashkenazi Jewish populations [109]. Respectively, they increase the risk of PD by ×4 and ×12. A number of meta-analyses have also estimated cumulative risks associated with all GBA mutations, but the significant heterogeneity of penetrance of individual *GBA* variants makes these estimates of limited usefulness [102,108]. Stratification of PD risk of *GBA* variants based on the Gaucher disease phenotype [namely the presence or absence of neurological features associated with them] provides a more relevant estimate of risk. Aggregated ‘mild’ variants increase PD risk by ×3 compared to the general population, whilst ‘severe’ variants (associated with neurological GD features) increase this risk by ×15 [110]. p.E326K (p.E365K) and p.T369M (p.T408M), found commonly in European populations, double PD risk compared to the general population, but do not cause Gaucher disease [108,111,112,113]. Collectively 10–15% of European PD patients carry a PD associated *GBA* variant, making it numerically the most significant PD genetic risk factor by some distance [33]. 

Similarly to *LRRK2,* both environmental and genetic cofactors are postulated to explain the reduced penetrance of *GBA* variants. A recent GWAS of individuals with *GBA* variants with and without PD showed that the PD risk loci implicating *SNCA* and *CTSB* were significantly associated with *GBA*-PD [114]. *CTSB* encodes a lysosomal protease which may interact with GCase. 

The closely related *GBAP* pseudogene has important implications for the study of *GBA*, as certain sequencing techniques are required to identify specific *GBA* variants. Depending on the techniques used, studies may only report on specifically targeted variants and miss other variants present in the *GBA* gene [115]. By using a PCR enrichment step, both Illumina based sequencing by synthesis [116]and long read sequencing using the Oxford Nanopore has been shown to reliably screen the entire *GBA* gene. The latter has been reliably able to phase variants, which is a distinct advantage, because quite a number of pathogenic *GBA* variants are in fact complex haplotypes derived from reciprocal translocations with the GBA pseudogene [117].

The clinical phenotype of *GBA*-PD is similar to that of idiopathic PD, although there is robust evidence that *GBA* variants in those with PD confer an earlier age of onset, higher rates of dementia and faster progression of motor symptoms [99,118,119,120,121]. ’Severe’ *GBA* variants are associated with more exaggerated clinical features [110]. This is also the case when variant pathogenicity is predicted with *in silico* methods [122]. Importantly, both p.E326K (p.E365K) and p.T369M(p.T408M), are associated with an increased risk of cognitive impairment and more rapid disease progression [123,124], in spite of the relatively small increase in PD risk associated with them.

In contrast to many of the other genes implicated in monogenic PD, the biological role of *GBA* is well understood. GCase is a lysosomal hydrolase that breaks down glucosylceramide into ceramide and glucose. *GBA* variants have differential effects on both the enzymatic activity of GCase and its trafficking within cells [125]. This identifies both enzyme action and cellular trafficking of GCase as potential therapeutic targets. Blood, cerebrospinal fluid (CSF) and brain GCase activity levels have been shown to be lower in idiopathic PD as well as *GBA*-PD, suggesting that novel *GBA* targeted therapies may also be beneficial in idiopathic PD [126,127,128].

#### 2.2.3. Heterozygous PRKN as a PD Genetic Risk Factor

Although bialleic *PRKN* variants are an established cause of PD, considerable controversy surrounds the effect on PD risk of heterozygous *PRKN* variants. Heterozygous *PRKN* variants are common finding amongst those with PD. One large study found that some 1.2% of early onset/familial PD cases carry a *PRKN* variant [33]. The same study found that *PRKN* carriers had an age at onset some 12 years lower than PD cases without other genetic risk factors. There is, however, conflicting data regarding the effect they exert on PD risk. A number of small studies suggest *PRKN* variants do increase PD risk however no association has been found in other studies [129] including in a recent analysis of some 2807 patients and 3627 controls [130,131,132]. It may be that only a portion of *PRKN* variants, and in particular those with a truncating effect on protein function, confer an effect of PD risk. This may explain the discontinuity of results seen.

## 3. Sporadic Parkinson’s Disease

In contrast to studies of familial PD that initially identified monogenic associations through studies of affected pedigrees, the genetics of sporadic PD has primarily advanced through case-control GWAS of common genetic variants. GWAS are powerful techniques to identify genetic loci that have a small but additive effect on traits of interest and can identify biological pathways relevant to that trait. The combined effect of multiple common genetic variants (typically defined as a minor allele frequency of greater than 5%) contributes to the risk of developing a common disease, such as sporadic PD. Whereas twin studies show greatest PD heritability when onset is below 50 years (capturing the highly penetrant but rare monogenic causes), GWAS heritability is greatest in those with PD onset over 50 years, demonstrating that common variants have the greatest genetic contribution to the risk of late-onset PD [9,17]. 

Table 2 summarises GWAS of idiopathic PD performed to date according to the National Human Genome Research Institute GWAS Catalog [133]. The majority of idiopathic PD-related GWAS have focused on variants associated with the risk of developing PD. Only a minority have interrogated possible associations with other traits such as age of PD onset, progression of disease or response to medications. Furthermore, most studies have used European cohorts thus limiting the applicability of results to worldwide populations. Table 2 demonstrates that the larger the GWAS cohort, the greater the number of significant loci identified. This has also been shown for other common continuous traits such as height and body mass index [134] and also other binary disease states such as type 2 diabetes [135]. The information from these GWAS can be utilised in two main ways: firstly, the biological functions captured by associated variants can identify underlying disease mechanisms; secondly, the combined information from all associated variants can produce a ‘polygenic risk score’ to quantify an individual’s genetic risk of developing a trait.

GWAS have identified new genes relevant to the risk of developing PD. However, the actual functional effect indicated by trait-associated variants can be challenging to determine. Moreover, significant variants tag a genomic region with which that variant is in linkage disequilibrium and most common variants are found in non-coding regions of the genome. Therefore, post-GWAS analyses are critical in assigning biological function to GWAS variants. Examples of post-GWAS analyses include pathway analysis (where groups of variants are analysed together to determine the relevance of sets of gene to the trait] and expression quantitative trait loci (eQTL) analysis (which identifies variants associated with the expression of genes and so identifies a regulatory element) [136]. In PD, robust associations from multiple GWAS and subsequent post-GWAS analyses have been identified for *SNCA*, *RAB29*, *MAPT*, *BST1*, *GAK*, *LRRK2* and *HLA-DRB5* amongst others [18,137]. *GAK* has a role in synaptic endocytosis and so links this pathway in sporadic PD to the rare forms of *DNAJC6* and *SYNJ1* monogenic PD. Intriguingly, some of these genes harbour common variants that increase risk to sporadic disease as well as rare variants that cause monogenic PD, indicating shared biological pathways (e.g., *SNCA*).

A polygenic risk score (PRS) allows the combined effect of GWAS variants to be quantified in an individual. A PRS will therefore depend on the variants included and will be specific to the cohort ethnicity and to the trait under consideration. Using the variants from Nalls et al. [18] there is approximately 4 times greater odds of PD in the highest PRS quartile compared with the lowest quartile. Additionally, the PD risk PRS has consistently been shown to correlate with age of onset in multiple ethnicities, despite the fact that PD age of onset GWAS have found very few significant variants [138,139,140,141]. A study using an Asian cohort demonstrated that an ethnicity-specific PRS enhances performance [142]. Despite this consistent association of PRS with disease state and age of onset, addition of PRS to clinical decision tools only has a limited impact [141,143]. A recent study used a novel approach to assess association between the PRS of genes within pre-specified functional pathways and the risk of PD and identified 46 significantly associated pathways, including 6 that did not involve any of the previously identified GWAS loci [144].

Despite this progress in identifying many common variants associated with PD risk, GWAS can still only account for 16–36% of PD heritability [18]. In the future, we anticipate that larger cohorts incorporating different ethnicities will identify more lower risk variants and that assessing different PD-related traits will further contribute to the polygenic architecture of PD. However it is important to emphasize that GWAS is of limited use for the identification of some genetic risk factors, for example *GBA*. Because it relies upon arrays of directly genotyped variants and then imputes other variants by linkage disequilibrium, important disease associated variants can be missed. In fact, GWAS was only able to identify the *GBA* locus using a candidate gene approach [145]. It may be that other such genetic risk factors contribute to PD risk but remain undetected by current GWAS analyses. Expanding our understanding of these genetic contributors to idiopathic PD risk is likely to require forensic examination of sequencing data, a time consuming, expensive and highly complex process [146]. 

## 4. Therapeutics

### 4.1. Disease-Modifying Agents for PD

A disease-modifying agent in PD would be one that slows or stops the processes leading to neuronal loss. Where there is a single gene abnormality that greatly increases the risk of PD, strategies targeted at that variant and its biological consequences are appealing. This is particularly the case for *GBA* and *LRRK2* because they are so much more common than other monogenic causes of PD. However, any successful agents may only be effective in that group of patients who harbour a variant and so applicability to idiopathic PD is not guaranteed. We summarise some of the therapies whose targets have been informed by PD genetics that are currently under evaluation for the disease-modification for PD.

#### 4.1.1. GBA

While Gaucher disease can be managed with enzyme replacement, GCase does not cross the blood brain barrier so this is not a viable option in PD [175]. Instead, small molecules that are able to cross the blood-brain barrier and enhance the action of GCase are being explored. Currently two agents are registered in phase II clinical trials. The first is ambroxol, which is a small molecule chaperone of GCase that is already in clinical use as a cough linctus medicine. It is well-tolerated and has been shown to increase levels of GCase protein in the CSF of 17 individuals with PD (8 with *GBA* variants) [176]. A blinded placebo-controlled trial is underway to assess whether ambroxol is able to improve cognition in PD dementia [NCT02914366] and is expected to complete in December 2021 [177], whilst a further phase III clinical trial evaluating the outcome in PD itself is planned. The second is venglustat, a small molecule inhibitor of glucosylceramide synthase which catalyses production of the substrate for GCase and has shown efficacy in mouse *GBA* and *SNCA* models of neurodegeneration [178]. Disappointingly, a phase II trial showed that although a reduction in glucosylceramide was demonstrated in CSF, no clinical difference was demonstrated comparted to controls (in fact, a non-significant deterioration was seen in the intervention arm) [179]. A third approach is the use of gene therapy, whereby a viral vector injected directly into CSF is used to restore the wildtype *GBA* sequence. The limitation of such approaches to date has been the inability to achieve sufficient transfection in the target organ. A phase I study is currently recruiting to evaluate PR001A, a viral vector-based therapy to deliver *GBA* which rescues GCase activity and reduces α-synuclein levels in cell and mouse models [180], in patients with moderate to severe PD and *GBA* variants (NCT04127578). Preliminary results evaluation are expected in mid-2021. 

#### 4.1.2. LRRK2

As already mentioned, inhibition of LRRK2 is an attractive therapeutic strategy as pathogenic variants cause overactivation of the kinase domain and loss of function variants in humans have no associated disease phenotypes. Moreover, a wide range of small molecule kinase inhibitors have been developed for use in other diseases [181]. Two molecules have emerged from pre-clinical testing that appear to offer suitable pharmacokinetic profiles and have completed phase I studies (NCT03710707, NCT04056689). The molecule DNL151 has recently been shown to be well tolerated in healthy volunteers and people with PD and demonstrated inhibition of LRRK2 function [182]. An alternative strategy targeting *LRRK2* overactivity is using antisense oligonucleotides to target mRNA for degradation. Intrathecal administration may also avoid off-target effects in other organs. A preclinical study in mice showed that centrally delivered *LRRK2* antisense oligonucleotide reduced α-synuclein aggregates and increased dopaminergic neurons, without effects on the kidneys or lungs [183]. A phase I study of a *LRRK2* antisense oligonucleotide (BIIB094) in people with PD is registered and due to complete in 2023 [NCT03976349].

#### 4.1.3. SNCA

As well as these studies specifically targeting *GBA* and *LRRK2* cellular processes, there are a range of compounds that target α-synuclein which could be beneficial in those with *SNCA* variants but also, importantly, those with idiopathic PD. Immunotherapy approaches attempt to remove toxic forms of α-synuclein using antibodies which are either passively administered or actively stimulated via vaccination. Two α-synuclein antibodies, prazinezumab and cinpanemab have progressed to phase II clinical trials (NCT03100149 and NCT03318523). The α-synuclein peptide mimic, PD01A, which stimulates an immune response has been shown to be well tolerated in a phase I study of people with PD [184]. A phase II trial is intended but not yet registered. As well as immunotherapy techniques, molecules that directly inhibit aggregation of α-synuclein are being evaluated. Phase II studies are registered for the molecules ANVS-401 (NCT04524351) and ENT-01 (NCT03781791). However, nilotinib, which is used in chronic myeloid leukaemia and has been shown to accelerate α-synuclein degradation, showed no efficacy [185]. 

#### 4.1.4. Mitochondria

Genetic and environmental evidence have implicated mitochondrial dysfunction in PD and strategies that enhance mitophagy are likely to be beneficial to all forms of PD. Three repurposed oral medications that improve mitochondrial function are being assessed in phase II trials of idiopathic PD – ursodeoxycholic acid (NCT03840005 [186] ), terazosin(NCT03905811) and nicotinamide (NCT03568968). In addition, the novel agent EPI-589 is being assessed in a phase II trial of both idiopathic PD and genetic forms of PD that alter mitochondrial function (NCT02462603). 

### 4.2. Pharmacogenomics

Genetics can allow for selection of treatments for genetically stratified disease subgroups. Both PD and diabetes manifest clinical features when the action of a chemical messenger (dopamine in PD and insulin in diabetes) is reduced. Diabetes therefore serves as a useful analogy for how our current knowledge of PD genetics may one day be used to optimise clinical care and management [187]. 

A major success in the field of diabetes genetics has been the recognition that certain monogenic causes of diabetes are best treated by specific treatments. In particular, the discovery that patients with activating variants in the *ABCC8* and *KCNJ11* [188] genes respond better to sulfonylurea medications than to insulin allowed individuals with these variants to stop insulin injections [189]. Maturity onset diabetes of the young (MODY) is another monogenic form of diabetes which presents in early adulthood and typically can be managed with oral hypoglycaemic medications. Genetic stratification of MODY subtypes has allowed for homogenous cohort studies and evidence that certain medications are preferable in specific gene variants [190,191]. Indeed, in the case of MODY due to *GCK* mutations, it has been possible to determine that no treatment at all is necessary [192]. Finally, the polygenic type 1 diabetes risk score can be used as one domain to help differentiate type 1 from type 2 diabetes and therefore determine whether an individual is likely to require insulin treatment [193].

In PD, choice of treatment depends on a range of factors including patient preference and risk of side effects. However, prediction of side-effects is difficult and some can have extremely serious consequences for an individual (e.g., impulse control disorders). The ability to accurately predict serious side effects, as well as likely response, would be of great benefit to individual patients. A large number of common variants in genes related to dopamine action and metabolism have been associated with medication effectiveness and side effects in PD. These include dopamine receptor genes (*DRD1*, *DRD2*, *DRD3*, *DRD4*), dopamine transporter gene (*SLC6A3*) and the catechol-O-methyltransferase gene (*COMT*) [194]. However, many of these association studies have been small in number and focus on a subset of candidate genes. Moreover, some have shown contradictory findings [195,196]. 

More recent studies have evaluated genome-wide common variants in an hypothesis-free method which have the potential to identify novel genetic modifiers of medication response [173]. Using genotype data in addition to clinical parameters has been shown to significantly improve the ability to predict the occurrence of impulse control disorders in people with a recent diagnosis of PD and a mean follow up of 2.2 years (AUC increased from 0.64 to 0.76). Indeed the heritability of impulse control disorder was estimated at 57%, suggesting a predominant genetic component [197]. In contrast, in a retrospective study of people with PD looking at the time to develop L-DOPA induced motor complications, addition of genotype to clinical data did not lead to any improvement in the model [198].

A recent systematic review of treatment in monogenic forms of PD highlights the challenges in drawing conclusions about the best treatment for each subtype [199]. Data were available for 2226 patients, with the majority being those with *PRKN* (1002) or *LRRK2* (820) variants. Overall 420 variants in 6 genes (*SNCA*, *LRRK2*, *VPS35*, *PRKN*, *PINK1*, *PARK7*) were identified. There were very high rates of missing data for doses and specific adverse effects. Overall the treatment efficacies and side-effects appear to be broadly similar to that of idiopathic PD but conclusions regarding rare variants and non-L-DOPA therapies should be treated with caution. Several groups have demonstrated poorer outcomes of deep brain stimulation surgery in those with Parkinson’s who carrying a *GBA* variant, with some suggesting that in these cases the benefits of surgery do not outweigh the risk and hence, other less invasive advanced PD therapies should be selected in these cases [200,201].

## 5. Selecting Appropriate PD Cohorts for Clinical Trials

Interventional clinical trial arms should be matched so that they only differ in terms of exposure to the intervention. Any difference, including genetic variation, between the arms can lead to false negative or false positive results. We know that some forms of monogenic PD progress at different rates (e.g., *GBA*-PD tends to have faster motor and cognitive progression then idiopathic PD). In addition, a higher PD risk PRS has been associated with faster motor progression as assessed by Hoehn & Yahr and UPDRS-III scores, which are commonly used as endpoints in clinical trials [202]. Indeed, in predictions of PD progression using machine learning models, genetic variation was a better predictor than imaging or CSF biomarkers [203]. 

Not accounting for genetic variation has the potential to inadvertently lead to mismatched clinical trial arms. A study of simulated clinical trial scenarios using genetic data from 5851 people with PD estimated that the average PRS varied by up 22% in trials containing up to 1000 individuals. Further analysis of virtual cohorts showed that for trials with 50 individuals and an intervention that reduced the MDS-UPDRS by 0.5/year, over one third would report false negative results as a result of genetically unbalanced arms [204]. Although these findings could be partially mitigated by larger sample sizes and longer follow ups, these strategies have implications in terms of cost and practicality. In principle, balancing clinical trial arms by genetics could lead to better evaluation of any intervention, although this would require baseline genotyping of all participants and appropriate consenting.

Recruitment of specific genetic risk factors for PD (for example only individuals with *LRRK2* p.G2019S PD) could enable more genetically stratified cohorts. This can also allow for targeted therapies to be studied as currently being recruited for *LRRK2*- and *GBA*-targeted compounds. However, we know that the PRS and other genetic factors still modify progression in these groups [114,205]. Conversely, as our understanding of the genetics underlying PD risk and progression evolves, balancing trial arms may become increasingly difficult, requiring an unattainably large pool of potential participants to draw from.

As well as balancing trial arms, there is potential for using genetics to recruit cohorts that progress faster, either because of monogenic risk or high PRS. Whereas for an individual the knowledge that certain groups progress faster on average may be disconcerting and unhelpful, for a clinical trial cohort this is useful information. Recruiting a cohort of *GBA* carriers, for instance, could reduce the follow up required for trials to meet their endpoints, thereby reducing cost, minimising participant burden and accelerating drug discovery [206]. Conversely, if indeed *LRRK2-*PD has a slower rate of progression, a larger trial size would be required.

### Prodromal PD

A major challenge in trials of all neurodegenerative conditions is recruiting individuals at an early stage of disease who have the most to gain from disease-modifying therapies [the so-called ‘window of opportunity’]. It has been suggested that a combination of clinical features and biomarkers can be utilised to identify people with pre-symptomatic or prodromal disease. PD has a well-established prodromal phase with published research criteria which, since the 2019 update, incorporates PRS and age-related penetrance of certain *GBA* and *LRRK2* variants [3]. This could potentially be utilised to identify those ‘at risk’ or with early PD who may respond more favourably to disease-modifying therapies. That said, the sensitivity and specificity of attempts to stratify the risk of conversion to PD in at risk populations have to date been disappointing [207,208,209], with the exception of those carrying *LRRK2* variants [210]. This may reflect the high penetrance [and by extension hazard ratio] associated with this genetic risk factor. 

A number of longitudinal cohorts have studied the pre-symptomatic features of PD and attempted to identify predictors of conversion to clinical PD, often using a combination of measures [211,212,213]. Both *LRRK2* and *GBA* carriers have been shown to exhibit prodromal PD features such as hyposmia [209], REM sleep behaviour disorder [214] and dopaminergic loss [215] and/or glial activation on PET imaging. This raises difficult ethical issues for clinical trials that still remain to be overcome. If an intervention is required to be taken for many years, then the safety profile of that intervention must be rigorously assessed. Given the range of penetrance risk and progression of PD even within specific variants, accurate power calculations and recruitment numbers are difficult to ascertain [216,217]. 

## 6. Concluding Remarks

Over the last 30 years it has been recognised that genetics contributes approximately 25% to the risk of developing PD. Characterisation of the exact genes involved and their function has enabled identification of relevant biological pathways are involved in its pathogenesis. This has been the case for both rare monogenic causes of PD and the more common late-onset polygenic PD. Indeed, there is significant overlap in the genetic risk factors suggesting shared disease mechanisms. With this increasing wealth of genetic knowledge, new strategies to assimilate the information and make it available to clinicians, researchers and patients are being created [218,219,220]. Whereas the treatment implications for different forms of monogenic diabetes have driven genetic testing into clinical practice, this is not yet the case for routine PD care, although examples of this are beginning to emerge [33,221,222]. While it is possible to provide some information to individual patients regarding risk of disease and prognosis, the range of predictions creates uncertainty. Knowledge of genetics has the potential to improve clinical trial design as well as to generate new and optimise existing therapeutic options for people with PD.

## Figures and Tables

**Table 1:** Summary of monogenic variants associated with Parkinson’s disease.

	Gene (HGNC Approved Name)	Alternative Gene Names	Inheritance	Pathogenicity	PD Phenotype	Function
High penetrance	*SNCA*	*PARK1*, *PARK4*, *NCAP*	AD	Pathogenic	Early-onset	Uncertain (encodes α-synuclein)
	*VPS35*	*PARK17, MEM3*	AD	Pathogenic	Typical	Retromer and endosomal trafficking
	*PINK1*	*PARK6*	AR	Pathogenic	Early-onset	Mitochondrial
	*PARK7*	*DJ-1*	AR	Pathogenic	Early-onset	
	PRKN	*PARK2*, *PARKIN*	AR	Pathogenic	Early-onset	
	*PLA2G6*	*PARK14, IPLA2*	AR	Pathogenic	Early-onset, atypical	Cell membrane
	*ATP13A2*	*PARK9*	AR	Pathogenic	Early-onset, atypical	Lysosomal
	*FBXO7*	*PARK15, FBX7*	AR	Pathogenic	Early-onset, atypical	Mitochondrial
	*POLG*	*POLG1, POLGA*	AD	Pathogenic	Early-onset, atypical	Mitochondrial DNA maintenance
	*DNAJC6*	*PARK19, DJC6*	AR	Likely pathogenic	Early-onset	Synaptic vesicle formation and trafficking
	*DNAJC13*	*PARK21, RME8*	AD	Conflicting reports	Typical	
	*TMEM230*	*C20ORF30*	AD	Conflicting reports	Typical	
	*SYNJ1*	*PARK20*	AR	Pathogenic	Early-onset, atypical	
	*VPS13C*	*PARK23*	AR	Pathogenic	Early-onset	Mitochondrial
	*CHCHD2*	*-*	AD	Pathogenic	Typical	Uncertain
	*DCTN1*	*-*	AD	Pathogenic	Atypical	Microtubule
Variable penetrance	*LRRK2*	*PARK8, DARDARIN*	AD	Pathogenic	Typical	Lysosomal, mitochondrial, microtubule
	*GBA*	*GBA1*	AD	Pathogenic	Typical	Lysosomal
Associated with PD but unlikely to be pathogenic	*HTRA2*	-	AD	Uncertain/likely benign	-	Mitochondrial
	*UCHL1*	*PARK5*	AD	Uncertain/likely benign	-	Ubiquitin-proteasome
	*GIGYF2*	*PARK11*	AD	Uncertain/likely benign	-	Uncertain
	*EIF4G1*	*-*	AD	Benign	-	mRNA translation
	*LRP10*	*LRP9*	AD ^1^	Uncertain	-	Uncertain

^1^ AD = autosomal dominant, AR = autosomal recessive, HNGC = HUGO Gene Nomenclature Committee.

**Table 2:** Summary of idiopathic Parkinson’s disease GWA studies included in NHGRI-EBI Catalog of human genome-wide association studies.

Study	Year	Cohort Size (Cases: Controls)	Trait	Ethnicity	Number of Genome-Wide Significant Loci
Fung et al. [147]	2006	267:270	PD	European	0
Pankratz et al. [148]	2009	857:867	Familial PD	European	0
Latourelle et al. [149]	2009	1604:440	PD age of onset	European	0
Satake et al. [150]	2009	1921:18274	PD	East Asian	4
Simón-Sánchez et al. [151]	2009	5074:8551	PD	European	3
Edwards et al. [152]	2010	1752:1745	PD	European	2
Hamza et al. [153]	2010	2000:1986	PD	European	4
Saad et al. [154]	2011	4271:9048	PD	European	2
Simón-Sánchez et al. [155]	2011	772:2024	PD	European	0
Liu et al. [156]	2011	2050:1836	PD	European	0
Spencer et al. [157]	2011	2744:7159	PD	European	3
Nalls et al. [158]	2011	12386:21026	PD	European	11
Do et al. [159]	2011	3426:29624	PD	European	8
Chung et al. [160]	2012	443:0	PD motor and cognitive outcomes	European	0
Hernandez et al. [161]	2012	387:496	Early-onset PD	Finnish	0
Pankratz et al. [145]	2012	7976:6350	PD	European	5
Davis et al. [162]	2013	31:767	PD	European	0
Hill-Burns et al. [163]	2014	4235:2782	Familial and sporadic PD	European	4
Nalls et al. [137]	2014	19061:100833	PD	European	24
Hu et al. [164]	2016	250:250	PD	East Asian	0
Hill-Burns et al. [165]	2016	1168:0	Familial PD age of onset	European	2
Siitonen et al. [166]	2017	403:1650	Early-onset PD	Finnish	13
Foo et al. [167]	2017	5904:30831	PD	East Asian	3
Chang et al. [168]	2017	26035:403190	PD	European	41
Wallen et al. [169]	2018	2676:0	PD age of onset	European	1
Blauwendraat et al. [170]	2019	28568:0	PD age of onset	European	2
Bandres-Ciga et al. [171]	2019	4783:3066	PD and PD age of onset	European	5
Nalls et al. [18]	2019	37688(plus 18618 proxy cases):1417791	PD	European	90
Ryu et al. [172]	2020	741:0	Motor complications of PD	East Asian	1
Cha et al. [173]	2020	200:0	PD motor response to zonisamide	East Asian	1
Tan et al. [174]	2020	2755:0	PD progression	European	1
Foo et al. [142]	2020	65257:1896188	PD	East Asian and European	10

## Data Availability

Data sharing not applicable.
